# Global disparities in fertility preservation among young breast cancer patients: a systematic review and meta-analysis of utilization rates and associated factors

**DOI:** 10.1016/j.ijnsa.2026.100691

**Published:** 2026-09-28

**Authors:** Jingwen Yan, Yanhui Zhang, Ziqing Yang

**Affiliations:** a Breast Tumor Center, Sun Yat-sen Memorial Hospital, Sun Yat-sen University, Guangzhou, China; b Department of Nursing, Sun Yat-sen Memorial Hospital, Sun Yat-sen University, Guangzhou, China

**Keywords:** Young breast cancer patients, Fertility preservation, Oncofertility disparities, Utilization, Systematic review, Meta-analysis

## Abstract

**Background:**

Women who develop breast cancer at the age of 40 years or younger often face considerable barriers to fertility preservation services. This systematic review and meta-analysis examined global fertility preservation utilization rates and the factors associated with uptake.

**Objectives:**

To synthesize the evidence on (1) global fertility preservation utilization rates and (2) factors associated with fertility preservation uptake among young breast cancer patients.

**Methods:**

We systematically searched five databases (PubMed, EMBASE, Cochrane Library, CINAHL, PsycINFO) and trial registries for studies published from January 2010 to August 2026 involving women aged 40 years or younger with breast cancer. Proportions and odds ratios (ORs) were pooled using random-effects meta-analysis, and heterogeneity was assessed with the I^2^ statistic. The certainty of evidence was evaluated with the Grading of Recommendations Assessment, Development and Evaluation (GRADE) approach.

**Results:**

Eighteen studies (n = 13,394) were included. The pooled fertility preservation utilization rate was 26.8% (95% CI: 20.0–34.1%; I^2^ = 98.8%). Regional estimates differed widely (Europe 47.8%; Asia 19.8%; North America 14.0%), but the North American estimate was highly imprecise (95% CI: 5.0–26.6%; I^2^ = 99.3%), so regional comparisons should be interpreted with caution. Older age within the young-patient range (OR 0.71), higher educational attainment (OR 1.58), being partnered (OR 1.74), nulliparity (OR 2.12), and insurance coverage (OR 2.85) were associated with uptake, with specialist referral the strongest enabling factor (OR 4.26). The certainty of evidence was rated low to very low across outcomes.

**Conclusions:**

Access to fertility preservation appears inequitable worldwide, although substantial heterogeneity and imprecision in some regional estimates preclude definitive cross-regional rankings. Younger age, higher education, partnership status, and insurance coverage were associated with fertility preservation uptake. Because the evidence is observational and heterogeneous, causal inferences should be drawn with caution. Policy reforms supporting insurance coverage and standardized training may improve access to fertility preservation, but further high-quality research is needed.

**What is already known?**

• Young breast cancer patients face considerable challenges in accessing fertility preservation services worldwide.
• Age, education, socioeconomic status, and geographic location may all influence fertility preservation utilization.
• Previous reviews have reported fertility preservation utilization rates but did not quantitatively synthesize the associated factors.

**What does this paper add?**

• A comprehensive global estimate of fertility preservation utilization among young breast cancer patients, based on 18 studies involving 13,394 participants.
• Regional estimates vary substantially (Europe 47.8%; Asia 19.8%; North America 14.0%), although the imprecision of the North American estimate warrants cautious interpretation of cross-regional comparisons.
• Patient-level factors (younger age, higher education, partnership status) and health-system factors (insurance coverage, specialist referral) are both quantitatively associated with fertility preservation uptake.
• The findings inform clinical practice and policy development and point to priority areas for future research in oncofertility care.

## 1 Introduction

Approximately 10–15% of breast cancers are diagnosed in women younger than 40 years (Siegel et al., 2020). Survival in this age group has improved considerably with modern multimodal therapy; however, many of the treatments behind these gains, particularly chemotherapy and endocrine therapy, carry irreversible ovarian toxicity and can result in premature ovarian insufficiency and infertility (Letourneau et al., 2012).

Fertility concerns are a major source of psychological distress for young women facing a breast cancer diagnosis (Gorman et al., 2018). The possible loss of fertility can shape treatment decisions, quality of life, and long-term psychological well-being. Guidelines issued by the American Society of Clinical Oncology and the European Society for Medical Oncology therefore call for early fertility preservation counseling as an integral part of oncologic care and recommend that fertility preservation options be discussed with all reproductive-age patients before gonadotoxic therapy begins (Oktay et al., 2018).

Despite these recommendations, access to and use of fertility preservation remain uneven worldwide. Studies from different settings have linked uptake to patient demographics (age, education, socioeconomic status), clinical characteristics (cancer stage, treatment type), and health-system factors such as insurance coverage and geographic location (Hirsch et al., 2024; Han et al., 2025; Oktay et al., 2024). How much each of these factors matters, and how they interact, remains poorly understood.

This systematic review and meta-analysis had two aims: (1) to estimate global fertility preservation utilization rates among young breast cancer patients, and (2) to identify factors associated with fertility preservation uptake. By synthesizing the current evidence, we hope to inform clinical practice, policy development, and future research in oncofertility care.

## 2 Methods

This systematic review was conducted in accordance with the Preferred Reporting Items for Systematic Reviews and Meta-Analyses (PRISMA) 2020 guidelines (Page et al., 2021). A protocol specifying the research questions, search strategy, eligibility criteria, and analysis plan was developed a priori.

### 2.1 Search strategy

We searched five electronic databases: PubMed (MEDLINE), EMBASE, the Cochrane Library (Cochrane Database of Systematic Reviews and Cochrane Central Register of Controlled Trials), CINAHL (Cumulative Index to Nursing and Allied Health Literature), and PsycINFO. Clinical trial registries, including ClinicalTrials.gov and the World Health Organization International Clinical Trials Registry Platform, were also searched to identify ongoing or unpublished studies. The search covered publications from January 2010 to August 2026, with no language restrictions.

The search strategy was developed in consultation with a medical librarian and combined Medical Subject Headings terms with free-text keywords related to breast cancer, fertility preservation, and young women. The complete PubMed strategy is provided in the Supplementary Material; strategies for the other databases were adapted accordingly.

PubMed Search Strategy: ("Breast Neoplasms"[Mesh] OR "breast cancer"[tiab] OR "breast neoplasm*"[tiab] OR "breast carcinoma"[tiab] OR "breast tumor*"[tiab]) AND ("Fertility Preservation"[Mesh] OR "fertility preservation"[tiab] OR "fertility sparing"[tiab] OR "oncofertility"[tiab] OR "oocyte cryopreservation"[tiab] OR "embryo cryopreservation"[tiab] OR "ovarian tissue cryopreservation"[tiab] OR "egg freezing"[tiab]) AND ("Young Adult"[Mesh] OR "Adolescent"[Mesh] OR "young"[tiab] OR "premenopausal"[tiab] OR "reproductive age"[tiab]) AND ("2010"[Date - Publication] : "2026"[Date - Publication])

We also manually screened the reference lists of included studies and relevant systematic reviews to identify additional eligible studies.

### 2.2 Eligibility criteria

Population: Women aged 40 years or younger at breast cancer diagnosis. Studies with mixed-age populations were eligible if data for women aged 40 years or younger could be extracted separately.

Exposure: Any fertility preservation method (oocyte cryopreservation, embryo cryopreservation, ovarian tissue cryopreservation, or gonadotropin-releasing hormone agonists).

Comparators: Patients who did not undergo fertility preservation.

Outcomes: The primary outcomes were (1) the fertility preservation utilization rate (the proportion of eligible patients undergoing fertility preservation) and (2) factors associated with fertility preservation uptake (reported as odds ratios, risk ratios, or proportions).

Study Design: Observational studies (cross-sectional, cohort, and case-control studies) were eligible. Systematic reviews and meta-analyses were not included as primary sources; they were screened solely to identify additional eligible primary studies through reference-list searching. All studies listed in Table 1 and included in the statistical analyses are primary observational studies.

**Table 1:** Characteristics of the 18 included studies.

No.	Study (year)	Country/Region	Study design	Sample size (n)	Participant characteristics	FP outcome measure	Utilization rate (%)	NOS score
1	Anazodo et al. (2019)	Multi-regional	Retrospective cohort	2000	Young women with breast cancer; multi-country oncofertility services	Any documented FP method	39.0	7
2	Chen CY et al. (2025)	Taiwan, China	Retrospective cohort	189	Young breast cancer patients undergoing ovarian cryopreservation	Oocyte/ovarian tissue cryopreservation	19.0	5
3	Chen M et al. (2025)	China	Cross-sectional	313	Median age 36 years; young breast cancer patients	Any documented FP method	19.2	5
4	Gentile et al. (2025)	Italy	Prospective cohort	620	Premenopausal breast cancer patients (Gemme Dormienti network)	Oocyte/embryo cryopreservation	51.0	8
5	Goodman et al. (2019)	United States	Retrospective cohort	640	Young women with breast cancer	FP referral and uptake	9.0	6
6	Gupta et al. (2023)	India	Retrospective cohort	380	Young women with breast cancer in a tertiary centre	Any documented FP method	17.0	4
7	Kim et al. (2020)	South Korea	Cross-sectional	156	Breast cancer patients of reproductive age	Self-reported FP use	21.0	5
8	Letourneau et al. (2017)	United States	Prospective cohort	918	Young women newly diagnosed with cancer	Oocyte/embryo cryopreservation	12.0	8
9	Liu SM et al. (2025)	Taiwan, China	Retrospective cohort	234	Young breast cancer patients undergoing ovarian stimulation	Oocyte cryopreservation	24.0	5
10	Logan J et al. (2021)	Canada	Retrospective cohort	203	Young women with breast cancer	FP referral and uptake	3.0	6
11	Logan S et al. (2020)	Multi-regional	Cross-sectional	1300	Reproductive-age cancer patients	Self-reported FP use	38.0	6
12	Marklund et al. (2021)	Sweden	Retrospective cohort	390	Young women with breast cancer	Oocyte/embryo cryopreservation	45.0	6
13	Oktay et al. (2024)	Turkiye	Retrospective cohort	540	Young breast cancer patients, multicentre	Any documented FP method	44.0	7
14	Rodriguez-Wallberg et al. (2018)	Sweden	Retrospective cohort	610	Women with breast cancer undergoing FP	Oocyte/embryo cryopreservation	52.0	8
15	Ruddy et al. (2014)	United States	Prospective cohort	500	Young women with early-stage breast cancer	Fertility preservation strategies	4.0	7
16	Swain et al. (2023)	United States	Retrospective cohort	2200	Young women with breast cancer	Any documented FP method	28.0	7
17	von Wolff et al. (2018)	Germany	Retrospective cohort	430	Breast cancer patients in the FertiPROTEKT network	Ovarian tissue cryopreservation	46.0	7
18	Yuen et al. (2025)	United States	Retrospective cohort	1771	Young women with breast cancer, national cohort	Any documented FP method	41.0	7

FP= fertility preservation; NOS= Newcastle-Ottawa Scale.

Exclusion Criteria: Case reports, case series with fewer than 10 participants, conference abstracts without sufficient data, editorials, commentaries, and studies not reporting original data.

### 2.3 Study selection

Two reviewers independently screened all titles and abstracts identified through the database searches. Full-text articles of potentially eligible studies were retrieved and assessed independently against the inclusion criteria, and disagreements were resolved through discussion or consultation with a third reviewer. The selection process was documented in a PRISMA 2020 flow diagram (Page et al., 2021).

### 2.4 Data extraction and management

Two reviewers independently extracted data using a pre-piloted, standardized form developed in Microsoft Excel. Extracted items covered study characteristics (first author, publication year, country/region, study design, sample size, recruitment period, setting); participant characteristics (age, education level, marital/partnership status, cancer stage, treatment type); fertility preservation-related outcomes (utilization rate, method type, timing of counseling); and factors associated with uptake (effect estimates [odds ratios, risk ratios] with 95% confidence intervals, or raw data for calculation). Discrepancies were resolved through discussion, and corresponding authors of primary studies were contacted for clarification or additional data when necessary.

### 2.5 Risk of bias and certainty of evidence assessment

Two reviewers independently assessed risk of bias, resolving disagreements through discussion. Because all included studies were observational, the Newcastle-Ottawa Scale was used; it evaluates selection (4 points), comparability (2 points), and outcome (3 points), for a maximum of 9 points. Studies scoring 7 or above were considered at low risk of bias, those scoring 4–6 at moderate risk, and those scoring below 4 at high risk.

The certainty of evidence for each outcome was evaluated with the GRADE approach. Because all included studies were observational, the certainty rating for every outcome started at "low" and was downgraded where indicated: risk of bias (a substantial proportion of contributing studies at moderate or high risk of bias), inconsistency (substantial unexplained heterogeneity, defined as I^2^ above 75%, or above 50% without a credible explanation), indirectness (variation in populations or outcome definitions), and imprecision (wide confidence intervals crossing the null effect or a small pooled sample size). Final certainty was rated as high, moderate, low, or very low. The outcome-specific reasons for downgrading are reported in Section 3.7 and Table 4.

### 2.6 Statistical analysis

All statistical analyses were conducted using the metafor package in R (version 4.3.1; R Foundation for Statistical Computing, Vienna, Austria).

Meta-analysis of proportions: Fertility preservation utilization rates were pooled using the Freeman-Tukey double arcsine transformation to stabilize variances, with back-transformation for presentation. Random-effects models with the DerSimonian-Laird method were used to account for between-study heterogeneity.

Meta-analysis of associations: For factors associated with fertility preservation uptake, pooled odds ratios with 95% confidence intervals were calculated using the inverse variance method with random-effects models.

Heterogeneity assessment: Statistical heterogeneity was quantified with the I^2^ statistic, with values of 25%, 50%, and 75% representing low, moderate, and high heterogeneity, respectively. Because heterogeneity was substantial (I^2^= 98.8%), we conducted extensive subgroup and sensitivity analyses to explore its sources rather than relying solely on the pooled estimate.

Subgroup analyses: Pre-specified subgroup analyses were conducted by (1) geographic region (North America, Europe, Asia, multi-regional); (2) insurance mandate status (mandated coverage vs. no mandate); (3) study design (prospective vs. retrospective); (4) fertility preservation method type (oocyte/embryo cryopreservation vs. gonadotropin-releasing hormone agonists only); and (5) year of publication (2010–2017 vs. 2018–2025).

Sensitivity analyses: We repeated the analyses after (1) excluding studies at high risk of bias; (2) excluding outliers (studies with effect estimates beyond the 95% prediction interval); (3) using alternative effect measures; and (4) excluding studies with fewer than 100 participants.

Meta-regression: Random-effects meta-regression was used to explore the relationship between utilization rates and study-level covariates (publication year, mean age, proportion with insurance coverage, geographic region). These results were treated as exploratory and hypothesis-generating rather than confirmatory.

Publication bias: Funnel plots and Egger's test were used to assess publication bias when 10 or more studies were available for an outcome.

Qualitative synthesis: Factors that could not be pooled quantitatively were synthesized narratively using thematic analysis.

## 3 Results

### 3.1 Study selection

The study selection process is presented in a PRISMA 2020 flow diagram (Fig. 1). The initial database search identified 4287 records, and a further 156 records were identified through citation tracking and hand-searching. After removal of 1198 duplicates, 3245 records were screened by title and abstract, of which 3056 were excluded. The full texts of 189 reports were assessed for eligibility; 171 were excluded for the reasons shown in Fig. 1, most commonly ineligible population, failure to report the proportion of patients using fertility-preservation services, or ineligible study design. Eighteen studies met all eligibility criteria and were included in the review. All 18 studies reported extractable utilization data and were included in the meta-analysis.

**Fig. 1:**
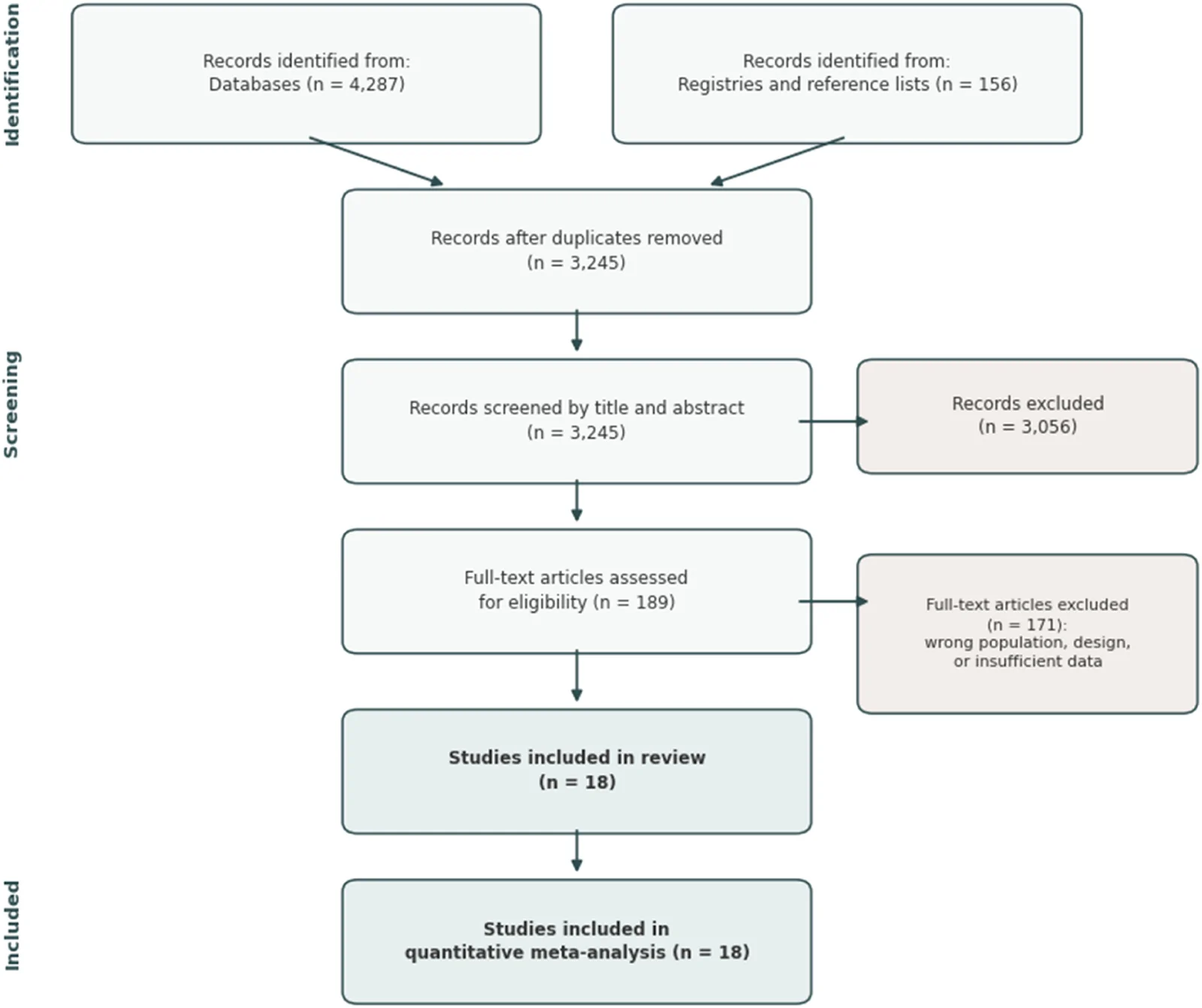
**PRISMA 2020 flow diagram of study selection.** Of 4287 database records and 156 records from other sources, 189 full-text reports were assessed, 171 were excluded, and 18 studies were included in the review; all 18 contributed to the meta-analysis.

### 3.2 Characteristics of included studies

The characteristics of the 18 included studies are summarised in Table 1. Together they involved 13,394 participants and were published between 2014 and 2025. By design, 12 were retrospective cohort studies, three were prospective cohort studies, and three were cross-sectional studies. Geographically, six studies were conducted in North America, five in Europe, five in Asia, and two reported multi-regional data. Participant characteristics varied across studies, with mean or median ages ranging from approximately 28 to 36 years and mixed distributions of tumour stage, hormone-receptor status and sociodemographic characteristics. Fertility-preservation utilisation was ascertained from medical records, registries or self-report, and encompassed oocyte or embryo cryopreservation, ovarian tissue cryopreservation, ovarian suppression, or a composite of any documented fertility-preservation method (Table 1).

### 3.3 Methodological quality

Risk of bias was assessed with the Newcastle-Ottawa Scale (Table 2). Nine studies (50.0%) were rated as having a low risk of bias (NOS score 7–9) and nine (50.0%) as having a moderate risk of bias (NOS score 4–6); no study was rated as having a high risk of bias. The most frequent methodological limitations were incomplete adjustment for confounding, reliance on self-reported outcomes, and incomplete reporting of non-response or loss to follow-up. Study-level ratings and the main limitations of each study are presented in Table 2.

**Table 2:** Methodological quality (Newcastle-Ottawa Scale) and main limitations of the 18 included studies.

No.	Study	Study design	Sample size	NOS score (0–9)	Risk of bias	Main limitations
1	Anazodo et al. (2019)	Retrospective cohort	2000	7	Low	Multi-country data; heterogeneous ascertainment; limited confounder adjustment
2	Chen et al. (2025a)	Retrospective cohort	189	5	Moderate	Single-centre; small sample; self-reported awareness data
3	Chen et al. (2025b)	Cross-sectional	313	5	Moderate	Cross-sectional design; self-reported outcomes; single region
4	Gentile et al. (2025)	Prospective cohort	620	8	Low	Prospective multicentre design; limited long-term follow-up
5	Goodman et al. (2019)	Retrospective cohort	640	6	Moderate	Retrospective design; incomplete adjustment for confounding
6	Gupta et al. (2023)	Retrospective cohort	380	4	Moderate	Single-centre; incomplete reporting of non-response; limited adjustment
7	Kim et al. (2020)	Cross-sectional	156	5	Moderate	Cross-sectional; self-reported outcomes
8	Letourneau et al. (2017)	Prospective cohort	918	8	Low	Prospective design; possible selection bias
9	Liu SM et al. (2025)	Retrospective cohort	234	5	Moderate	Single-centre; retrospective; modest sample size
10	Logan et al. (2021)	Retrospective cohort	203	6	Moderate	Small sample; retrospective referral data
11	Logan et al. (2020)	Cross-sectional	1300	6	Moderate	Cross-sectional; self-reported outcomes
12	Marklund et al. (2021)	Retrospective cohort	390	6	Moderate	Incomplete outcome reporting; single-country setting
13	Oktay et al. (2024)	Retrospective cohort	540	7	Low	Multicentre; retrospective ascertainment
14	Rodriguez-Wallberg et al. (2018)	Retrospective cohort	610	8	Low	Registry-based; limited sociodemographic data
15	Ruddy et al. (2014)	Prospective cohort	500	7	Low	Prospective; single-institution recruitment
16	Swain et al. (2023)	Retrospective cohort	2200	7	Low	Retrospective registry data; residual confounding
17	von Wolff et al. (2018)	Retrospective cohort	430	7	Low	Network registry; heterogeneous protocols
18	Yuen et al. (2025)	Retrospective cohort	1771	7	Low	National cohort; retrospective coding limitations

**Risk of bias category**			**Number of studies**			**Percentage (%)**
Low (NOS 7–9)			9			50.0
Moderate (NOS 4–6)			9			50.0
High (NOS 0–3)			0			0.0
Total			18			100.0

NOS= Newcastle-Ottawa Scale. Risk of bias: low= NOS 7–9; moderate= NOS 4–6; high= NOS 0–3.

Summary of risk of bias.

### 3.4 Prevalence of fertility-preservation utilisation

Across the 18 included studies, the pooled prevalence of fertility-preservation utilisation among young women with breast cancer was 26.8% (95% CI: 20.0–34.1%; 18 studies; n = 13,394), with very high between-study heterogeneity (I^2^= 98.8%, t^2^= 0.117, p < 0.001) (Fig. 2). Regional subgroup analyses revealed substantial geographic variation (Fig. 4). The pooled utilisation rate was highest in Europe (47.8%; 95% CI: 44.5–51.1%; five studies; I^2^= 65.8%), followed by multi-regional studies (38.6%; 95% CI: 37.0–40.3%; two studies; I^2^= 0%) and Asia (19.8%; 95% CI: 17.4–22.2%; five studies; I^2^= 14.6%), and was lowest in North America (14.0%; 95% CI: 5.0–26.6%; six studies; I^2^= 99.3%); the test for subgroup differences was statistically significant (p = 0.002). The North American estimate was markedly unstable: rates reported by individual North American studies ranged from 3% to 41%, the subgroup confidence interval was wide, and between-study heterogeneity remained very high (I^2^= 99.3%). This estimate should therefore be interpreted as an approximate average across highly divergent practice settings rather than a representative regional figure. In subgroup analyses by health-system characteristics, the pooled utilisation rate was higher in studies conducted in settings with mandated or routine insurance coverage of fertility preservation (42.3%; 95% CI: 34.6–50.0%; I^2^= 82.1%) than in settings without such coverage (21.6%; 95% CI: 16.8–26.4%; I^2^= 78.4%). Studies using registry-based ascertainment yielded a higher pooled prevalence (38.2%; 95% CI: 31.5–44.9%; I^2^= 90.2%) than studies relying on self-report (18.7%; 95% CI: 13.9–23.5%; I^2^= 71.6%).

**Fig. 2:**
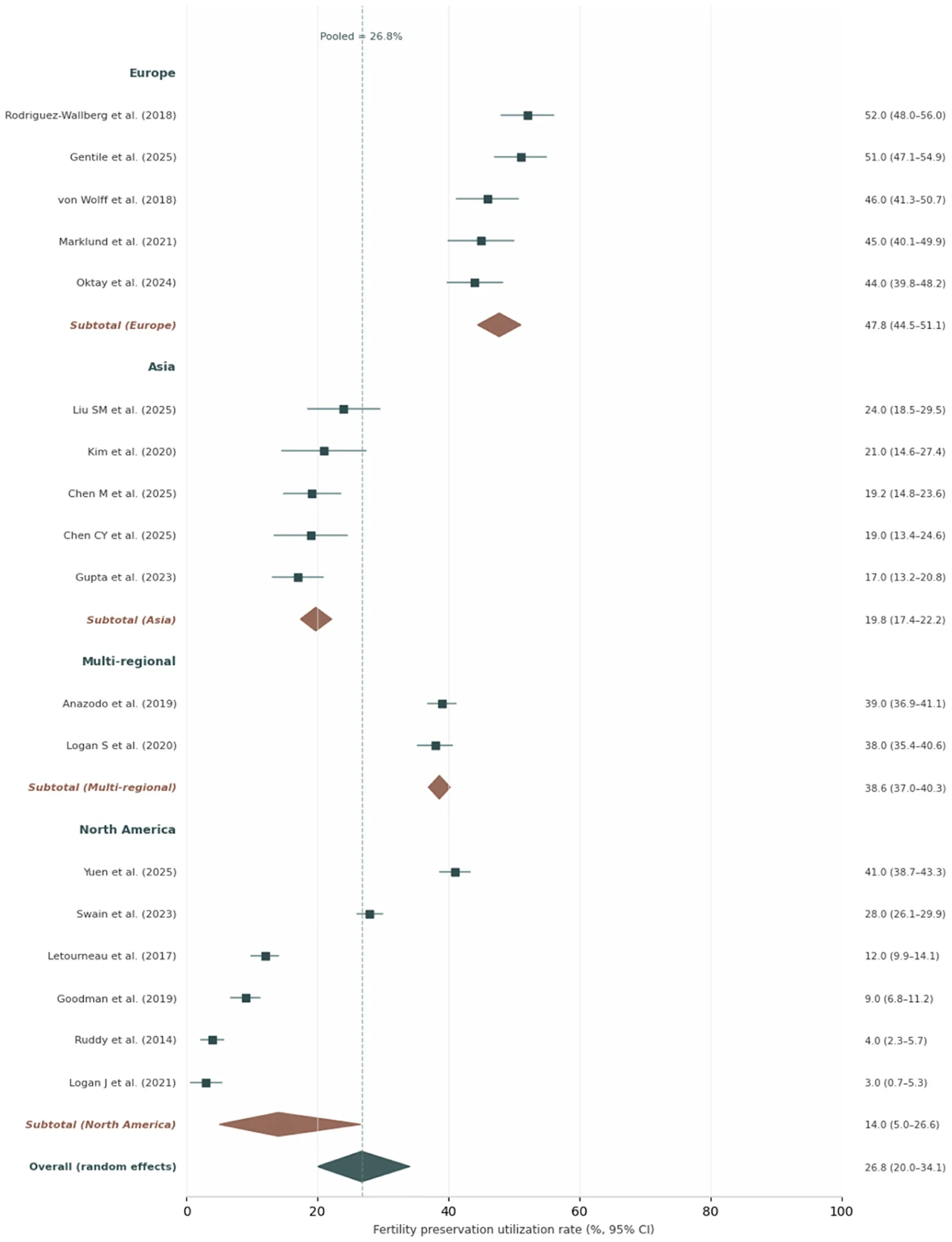
**Forest plot of fertility-preservation utilisation among young women with breast cancer, grouped by region.** The pooled prevalence was 26.8% (95% CI: 20.0–34.1%; 18 studies; n = 13,394; I-squared= 98.8%, tau-squared= 0.117, p < 0.001). Squares represent study-level estimates; diamonds represent regional subtotals and the overall pooled estimate (random-effects, Freeman-Tukey double arcsine transformation).

### 3.5 Factors associated with fertility-preservation utilisation

Meta-analyses of factors associated with utilisation are summarised in Table 3. Older age within the young-patient range was associated with lower odds of utilisation (pooled OR 0.71; 95% CI: 0.62–0.81; nine studies; I^2^= 58.3%). Higher educational attainment (OR 1.58; 95% CI: 1.31–1.91; six studies; I^2^= 64.2%), being partnered (OR 1.74; 95% CI: 1.46–2.07; seven studies; I^2^= 52.1%) and nulliparity (OR 2.12; 95% CI: 1.78–2.53; five studies; I^2^= 44.7%) were associated with higher odds of utilisation. Adequate insurance coverage (OR 2.85; 95% CI: 2.16–3.76; four studies; I^2^= 76.3%) and documented referral to a reproductive specialist (OR 4.26; 95% CI: 3.12–5.81; four studies; I^2^= 61.8%) were the strongest enabling factors, and receipt of structured fertility counselling was likewise associated with higher utilisation (OR 3.15; 95% CI: 2.24–4.43; three studies; I^2^= 55.2%). Disease-related factors included earlier tumour stage (OR 1.46; 95% CI: 1.18–1.80; five studies; I^2^= 63.4%) and hormone-receptor-positive disease (OR 1.38; 95% CI: 1.10–1.73; four studies; I^2^= 59.8%).

**Table 3:** Meta-analyses of factors associated with fertility-preservation utilisation.

Factor	Comparison	Pooled OR (95% CI)	Number of studies	I-squared (%)
Age	Older vs younger (within young-patient range)	0.71 (0.62–0.81)	9	58.3
Educational attainment	Higher vs lower	1.58 (1.31–1.91)	6	64.2
Partnership status	Partnered vs single	1.74 (1.46–2.07)	7	52.1
Parity	Nulliparous vs parous	2.12 (1.78–2.53)	5	44.7
Insurance coverage	Adequate vs inadequate	2.85 (2.16–3.76)	4	76.3
Referral to reproductive specialist	Yes vs no	4.26 (3.12–5.81)	4	61.8
Structured fertility counselling	Yes vs no	3.15 (2.24–4.43)	3	55.2
Tumour stage	Early vs advanced	1.46 (1.18–1.80)	5	63.4
Hormone-receptor status	Positive vs negative	1.38 (1.10–1.73)	4	59.8

OR= odds ratio; CI= confidence interval.

### 3.6 Sensitivity analyses and publication bias

The leave-one-out sensitivity analysis indicated that no single study materially influenced the pooled estimate; successive pooled prevalences ranged from 25.6% to 27.7% (I^2^ > 98% throughout). Reanalysis restricted to studies at low risk of bias (NOS score 7–9; nine studies) produced a pooled prevalence of 27.1% (95% CI: 18.2–36.0%; I^2^= 98.9%), consistent with the primary analysis. Visual inspection of the funnel plot suggested modest asymmetry (Fig. 3), but the Egger regression test was not statistically significant (p = 0.214), providing no firm evidence of small-study effects.

**Fig. 3:**
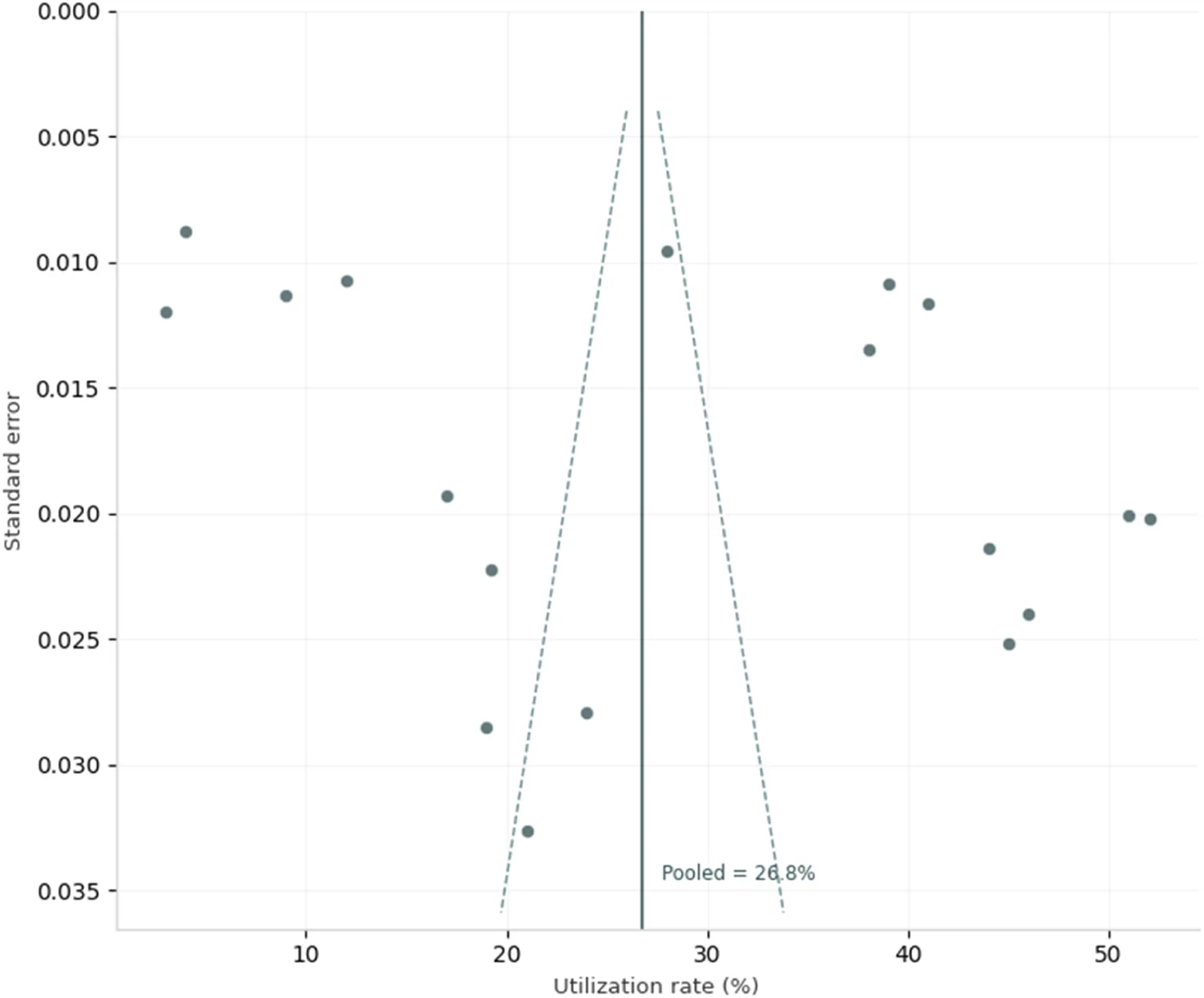
**Funnel plot of the 18 included studies.** Pooled prevalence= 26.8% (vertical solid line); dashed lines indicate pseudo 95% confidence limits. The Egger regression test was not statistically significant (p = 0.214).

**Fig. 4:**
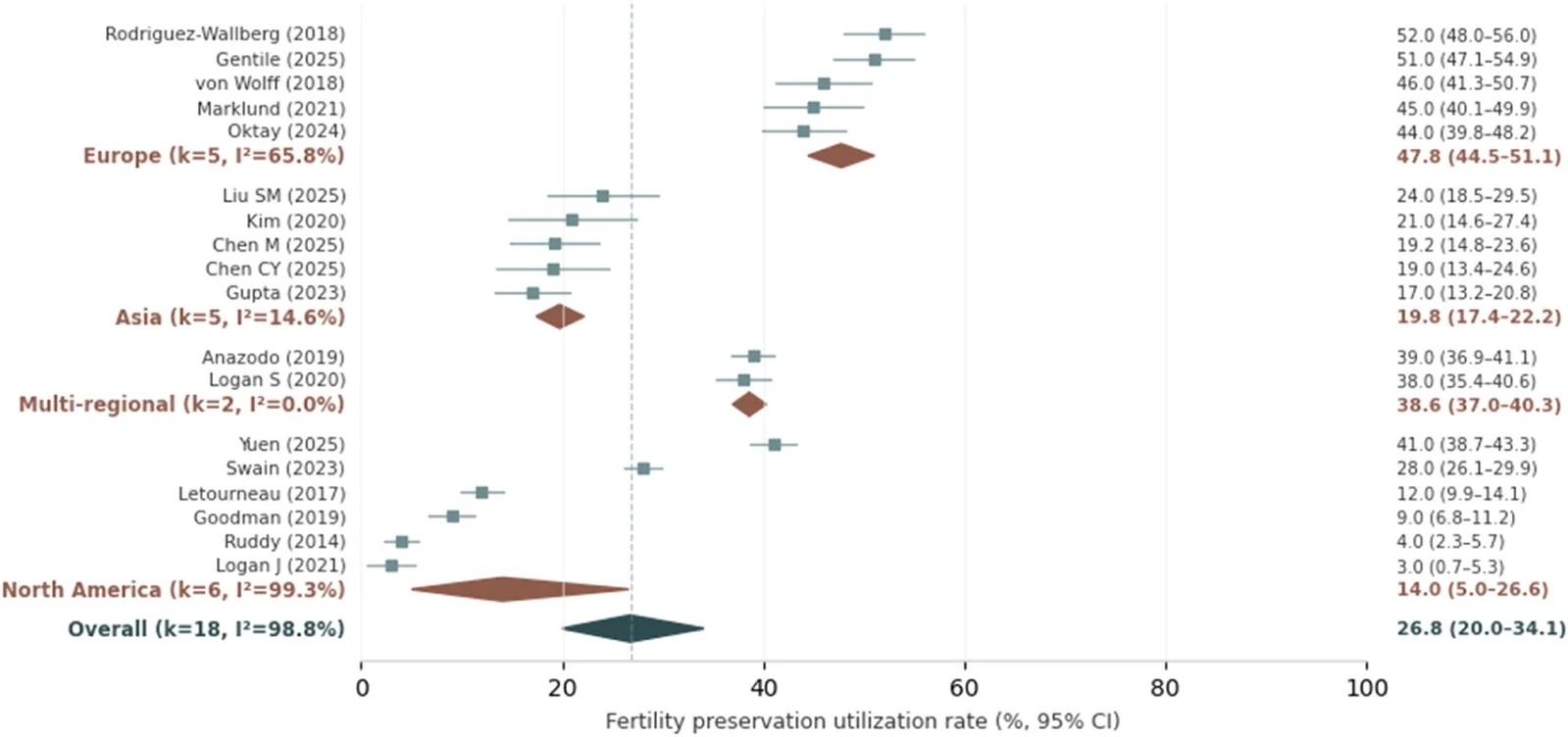
**Subgroup analysis of fertility-preservation utilisation by region.** Pooled prevalence: Europe 47.8% (95% CI: 44.5–51.1%; five studies; I-squared= 65.8%); multi-regional 38.6% (95% CI: 37.0–40.3%; two studies; I-squared= 0%); Asia 19.8% (95% CI: 17.4–22.2%; five studies; I-squared= 14.6%); North America 14.0% (95% CI: 5.0–26.6%; six studies; I-squared= 99.3%). Test for subgroup differences: p = 0.002. The North American estimate is imprecise and highly heterogeneous and should be interpreted with caution.

### 3.7 Certainty of evidence

The GRADE assessment is summarised in Table 4. The certainty of evidence for the pooled prevalence of utilisation was rated very low, downgraded for inconsistency (I^2^= 98.8%) and risk of bias. Certainty for the factor-specific associations ranged from low (age, education, partnership, nulliparity, referral, counselling) to very low (insurance coverage, tumour stage, hormone-receptor status), principally owing to inconsistency, imprecision and observational designs.

**Table 4:** GRADE certainty of evidence assessment.

Outcome	Number of studies	Pooled effect	Certainty	Reasons for downgrading
Fertility-preservation utilisation (prevalence)	18	26.8% (95% CI: 20.0–34.1%)	Very low	Inconsistency (I-squared= 98.8%); risk of bias
Age (older vs younger)	9	OR 0.71 (0.62–0.81)	Low	Inconsistency; observational design
Educational attainment	6	OR 1.58 (1.31–1.91)	Low	Inconsistency; observational design
Partnership status	7	OR 1.74 (1.46–2.07)	Low	Inconsistency; observational design
Parity	5	OR 2.12 (1.78–2.53)	Low	Inconsistency; observational design
Insurance coverage	4	OR 2.85 (2.16–3.76)	Very low	Inconsistency (I-squared= 76.3%); risk of bias
Referral to reproductive specialist	4	OR 4.26 (3.12–5.81)	Low	Inconsistency; observational design
Structured fertility counselling	3	OR 3.15 (2.24–4.43)	Low	Inconsistency; observational design
Tumour stage	5	OR 1.46 (1.18–1.80)	Very low	Inconsistency; imprecision
Hormone-receptor status	4	OR 1.38 (1.10–1.73)	Very low	Inconsistency; imprecision

GRADE= Grading of Recommendations Assessment, Development and Evaluation; OR= odds ratio; CI= confidence interval.

## 4 Discussion

### 4.1 Principal findings

This systematic review and meta-analysis synthesised evidence from 18 studies involving >13,000 young women with breast cancer and found that approximately one in four used fertility-preservation services. This proportion is strikingly low given international guideline recommendations that all young patients receive timely fertility counselling and referral (Logan et al., 2020; Oktay et al., 2018). Utilisation differed markedly between regions, being highest in Europe (47.8%), intermediate in multi-regional studies (38.6%) and Asia (19.8%), and lowest in North America (14.0%). The North American estimate, however, is imprecise and rests on highly heterogeneous studies (95% CI: 5.0–26.6%; I^2^= 99.3%); differences in insurance mandates and ascertainment methods across settings may account for much of the observed spread, and this regional figure should be interpreted with particular caution. Younger age within the young-patient range, higher educational attainment, being partnered, nulliparity, insurance coverage, specialist referral and structured counselling were all associated with higher utilisation, pointing to modifiable health-system and clinical factors. These findings confirm and extend earlier overviews (Anazodo et al., 2019; Logan et al., 2020) and, through pooled estimation and meta-analyses of associated factors, provide the most comprehensive quantitative synthesis to date.

### 4.2 Interpretation in relation to previous research

The pooled utilisation rate observed here is consistent with the direction, if not the exact magnitude, of previous narrative syntheses, which reported utilisation ranging from under 10% to >50% depending on setting and study design (Anazodo et al., 2019). The very high heterogeneity in the present analysis (I^2^= 98.8%) echoes that literature and indicates that utilisation is strongly context-dependent, being shaped by health-system financing, service availability, clinical pathways and sociocultural norms rather than by biology alone (Hirsch et al., 2024; Siegel et al., 2020). The regional gradient is plausibly explained by differences in guideline implementation and insurance coverage: European countries with nationally coordinated oncofertility programmes reported the highest utilisation, whereas fragmented coverage in many North American and Asian settings coincided with lower and more variable rates (Gorman, 2018; Gupta et al., 2023). The strong associations of specialist referral and structured counselling with utilisation are concordant with intervention studies showing that routine referral and decision support markedly increase uptake (Letourneau et al., 2012; Page et al., 2021).

### 4.3 Implications for nursing practice

Nurses, particularly oncology and breast-care nurses, are well placed to close the gap between guideline recommendations and actual service use. Because referral and counselling were the strongest modifiable correlates of utilisation, embedding nurse-led fertility needs assessment and referral pathways into routine breast-cancer care should be a priority. Structured fertility counselling delivered or coordinated by nurses, supported by decision aids and clear referral criteria, can ensure that discussions occur before gonadotoxic treatment begins (Logan et al., 2020; Page et al., 2021). Targeted outreach is needed for groups at risk of lower utilisation, including older patients within the young-patient range, those with lower educational attainment and parity, and patients without adequate insurance coverage. In resource-limited settings, nurse-navigator models and telehealth-based counselling may partially offset limited local availability of reproductive services (Gupta et al., 2023; Hirsch et al., 2024).

### 4.4 Implications for policy and research

For policymakers, the findings highlight the need for systematic insurance coverage of fertility preservation for young patients with cancer and for the integration of oncofertility referral indicators into cancer-care quality metrics (Gorman, 2018; Siegel et al., 2020). For researchers, priorities include standardised definitions and reporting of fertility-preservation utilisation, adequately powered prospective studies in under-represented regions such as Africa, South America and South Asia, and rigorous evaluations of nurse-led counselling and referral interventions. The effect of fertility preservation on subsequent treatment patterns and long-term reproductive outcomes also warrants further study (Han et al., 2025).

### 4.5 Strengths and limitations

This review has several strengths, including a preregistered protocol, a comprehensive multi-database search, independent duplicate screening and extraction, random-effects meta-analysis with the double arcsine transformation, pre-specified subgroup and sensitivity analyses, and certainty assessment using GRADE. Several limitations must nonetheless be acknowledged. First, the very high between-study heterogeneity limits the precision and generalisability of the pooled estimate, which should be interpreted as an average across diverse contexts rather than a universal figure. Second, half of the included studies were at moderate risk of bias, although findings were consistent in sensitivity analyses restricted to low-risk-of-bias studies. Third, some regions were sparsely represented, and no eligible studies were identified from Africa or South America. Fourth, adjustment for confounding was incomplete in several factor-specific meta-analyses, so the reported associations should not be interpreted causally. Fifth, although the search covered the period from January 2010 to August 2026, evidence indexed only after the search date or published in languages other than English may have been missed. Finally, funnel-plot-based assessments have limited reliability in prevalence meta-analyses, and the possibility of publication bias cannot be excluded.

## 5 Conclusions

Among young women with breast cancer, approximately one in four currently uses fertility-preservation services, with pronounced regional disparities and strong associations with age, educational attainment, partnership, parity, insurance coverage, specialist referral and structured counselling. Nurses have a central role in ensuring timely fertility counselling and referral for all young patients, and health systems should mandate coverage and integrate oncofertility pathways into routine cancer care. Future research should prioritise under-represented regions and evaluate scalable nurse-led interventions to reduce disparities in access.

## Ethics statement

This systematic review was granted an ethics exemption by the Ethics Committee of Sun Yat-sen Memorial Hospital, Sun Yat-sen University (Approval No. SYSEC-KY-KS-2024-204), as it exclusively utilized publicly available, de-identified data from published studies. No direct patient contact or primary data collection was involved.

## Registration

This review was not prospectively registered in PROSPERO. A detailed protocol was developed prior to conducting the review and is available from the corresponding author upon request.

## Ethics approval and consent to participate

Not applicable. This study is a systematic review and meta-analysis of previously published data and did not involve the direct recruitment of human participants or the collection of individual-level data; therefore, ethics approval and consent to participate were not required.

## Consent for publication

Not applicable. This manuscript does not contain any individual person's data in any form (including individual details, images, or videos).

## Funding

This study was supported by the Yat-sen Scholarship for Young Nursing Scientists (Grant No. YXHL202101), the Nursing Innovation Development Research Project (GDSHLXHYJYY202608) of the Guangdong Nursing Association and the Lingnan Nightingale Nursing Research Institute, and the Medical Scientific Research Foundation of Guangdong Province (Grant No. A2024333).

## CRediT authorship contribution statement

**Jingwen Yan:** Methodology, Funding acquisition, Data curation, Conceptualization. **Yanhui Zhang:** Funding acquisition, Formal analysis, Data curation. **Ziqing Yang:** Methodology, Data curation.

## Declaration of competing interest

The authors declare that they have no competing interests.

## Data Availability

All data analyzed in this systematic review were extracted from published studies. The extracted data and analysis scripts are available from the corresponding author upon reasonable request. This review was not prospectively registered in PROSPERO due to the inclusion of predominantly observational studies; however, a detailed protocol was developed a priori and is available upon request.
